# Editorial Strabismus

**Published:** 1902-01

**Authors:** 


### EDITORIAL STRABISMUS.

Our gentle contemporary, the *Country Gentleman*, has published a paragraph which is quoted with apparent approval by the *Jersey Bulletin* (because the *Jersey Bulletin* does not print anything it does not approve unless to get an opportunity to give it a dig), to the effect that a certain Brooklyn physician has inoculated a woman (who presented herself voluntarily for the experiment) with tubercle bacilli from a cow, and has produced tuberculosis in his subject. The *Country Gentleman* inquires: If this experiment terminates fatally, why should not the Brooklyn physician be conducted to the electric chair, there to expiate his crime?

Without attempting an answer to the above question we submit that the following are worthy of consideration, and we refer them, with our compliments, to the *Country Gentleman* and the *Jersey Bulletin*:

If tubercle bacilli from cattle are capable of producing tuberculosis in man, is it any worse to inoculate one voluntary subject in the shoulder than it is to inoculate a large number of involuntary subjects through the digestive tract?

If the experiment cited results fatally, and the suggestion of the *Country Gentleman* as to the treatment of the Brooklyn physician is carried out, what should be done with the editors of agricultural papers who have advised and encouraged the feeding of tubercle-producing germs to children?

